# Phenotyping Key Fruit Quality Traits in Olive Using RGB Images and Back Propagation Neural Networks

**DOI:** 10.34133/plantphenomics.0061

**Published:** 2023-06-23

**Authors:** Giuseppe Montanaro, Angelo Petrozza, Laura Rustioni, Francesco Cellini, Vitale Nuzzo

**Affiliations:** ^1^ Università degli Studi della Basilicata, 85100 Potenza, Italy.; ^2^ ALSIA, Agenzia Lucana Sviluppo Innovazione in Agricoltura, Metapontum Agrobios Research Center, 75010 Metaponto, Italy.; ^3^ Department of Biological and Environmental Sciences and Technologies, University of Salento, Lecce, Italy.

## Abstract

To predict oil and phenol concentrations in olive fruit, the combination of back propagation neural networks (BPNNs) and contact-less plant phenotyping techniques was employed to retrieve RGB image-based digital proxies of oil and phenol concentrations. Fruits of cultivars (×3) differing in ripening time were sampled (~10-day interval, ×2 years), pictured and analyzed for phenol and oil concentrations. Prior to this, fruit samples were pictured and images were segmented to extract the red (R), green (G), and blue (B) mean pixel values that were rearranged in 35 RGB-based colorimetric indexes. Three BPNNs were designed using as input variables (a) the original 35 RGB indexes, (b) the scores of principal components after a principal component analysis (PCA) pre-processing of those indexes, and (c) a reduced number (28) of the RGB indexes achieved after a sparse PCA. The results show that the predictions reached the highest mean *R*^2^ values ranging from 0.87 to 0.95 (oil) and from 0.81 to 0.90 (phenols) across the BPNNs. In addition to the *R*^2^, other performance metrics were calculated (root mean squared error and mean absolute error) and combined into a general performance indicator (GPI). The resulting rank of the GPI suggests that a BPNN with a specific topology might be designed for cultivars grouped according to their ripening period. The present study documented that an RGB-based image phenotyping can effectively predict key quality traits in olive fruit supporting the developing olive sector within a digital agriculture domain.

## Introduction

Olive oil is increasingly used in the human diet as a functional food due to its healthy antioxidants (e.g., phenolic compounds) [1] contributing to the globally expanding olive crop [2]. In addition, phenolic compounds (phenols) share about 50% of oil antioxidant power, contributing to its stability over time [3].

Despite these relevant features of phenols, the concentration of oil (% fresh weight) is the prominent quality trait generating the overall profitability of the crop. In contrast with this, to summarize the intrinsic quality of fruit, the seasonal pattern of both oil and phenol concentrations should be accounted for. Importantly, these patterns dynamically change during the fruit growing season following a roughly linear or parabolic trend in oil and phenols, respectively [2,4]. Hence, along with management practices, harvest time influences oil and phenol abundance and in turn yield quality and crop profitability. Hence, several fruit ripening indexes have been proposed to destructively track the maturity process and suggest the optimal harvest time in olives including fruit detachment force, skin pigmentation, and flesh firmness [5,6].

However, the determination of these indexes involves several limitations (e.g., the high consumption of time and high labor cost) when compared to those falling within a digital agriculture domain. For example, employing noncontact image-based methods is expanding to support crop management to face various issues including stress conditions, nutrition, water management, and targeting fruit quality [7,8]. Accordingly, in the olive sector, several studies have focused on image-based methods to develop fruit maturity indexes mainly for fruit classification purposes after harvest [9,10]. However, imaging within a regression context to track the variation of olive skin color associated with changes in quality trait(s) has yet to be adequately explored.

Plant phenomics is an innovative, non-invasive, image-based technology that is still being developed, through which it is possible to identify plant features and retrieve their quantitative responses to various stimuli. Within an affordable image-based plant phenotyping, the easily accessible red (R), green (G), and blue (B) images are frequently used as a proxy of physiological traits [11,12]. Adoption of RGB images, which also have a relatively low cost, might be in favor of extensive exploitation of plant phenotyping considering the wide diffusion of RGB cameras even on smartphones [13]. Along with the diffusion of RGB-based imaging, changes of color have been associated with changes in plant/organ status.

For example, changes in the green color of olive fruit during ripening have been associated with a change in chlorophyll concentration, and the fraction of green olives at harvest influences olive oil quality [14]. There are several RGB-based indexes estimating the chlorophyll concentration in leaf and fruit tissues in wheat and rye [15], in sugar beet leaves [16], and in citrus skin to monitor its ripening [17]. A set of RGB-based indexes has been employed by Zakaluk and Ranjan [18] to monitor plant water status in potato leaf. Furthermore, the amount of water in olive fruit is inversely correlated to its oil content throughout fruit ripening [19]. Hence, using such water-related RGB indexes would be reasonable to use to estimate the amount of oil in fruit. Recently, RGB images have been used along with non-destructive methods for quantifying total polyphenols in the bark of *Calycophyllum spruceanum* [20] and in olive fruit [21]. Hence, within this context, the association of polyphenols and RGB indexes in olive fruit is reasonable.

Along with the increasing use of imaging, it should be noted that it is increasingly combined with artificial neural networks (ANNs) to improve the prediction (classification and regression) of plant traits [9,10,22,23]. For example, the back propagation neural network (BPNN) is an ANN model with an adaptive and self-learning function particularly useful to solve nonlinear problems in plant science fields (e.g., fruit quality) [24,25]. However, ANN might suffer uncertainties including overfitting and reduced (or no) convergence, which might also be related to the network topology and redundant input variables [18]. Hence, the combination of ANN with principal component analysis (PCA) for extracting new features or selecting relevant variables is suggested to control the overfitting issue [26]. In addition, to further improve the interpretability of the PCA results (i.e., loadings) to support the BPNN topology design, an increased sparsity of loadings might be achieved employing a genetic algorithm (GA) and sparse PCA [27].

Taking into account this background, the present study tested the hypothesis that oil and phenol concentrations are predictable throughout the season by a BPNN fed with RGB-based colorimetric indexes retrieved through imaging. To test this hypothesis, olive samples from field-grown olive cultivars (×3) were sampled (×2 years) for imaging to extract R, G, and B mean pixel values and for analytical determinations of oil and phenol concentrations. The second objective was to compare the performance accuracy of 3 BPNNs using as input variables (a) a set of RGB-based colorimetric indexes, (b) the scores of principal components (PCs) after a PCA pre-processing of those indexes, and (c) a reduced number of the RGB indexes as defined after a sparse PCA.

## Materials and Methods

### Experimental design

**Fig. 1.**
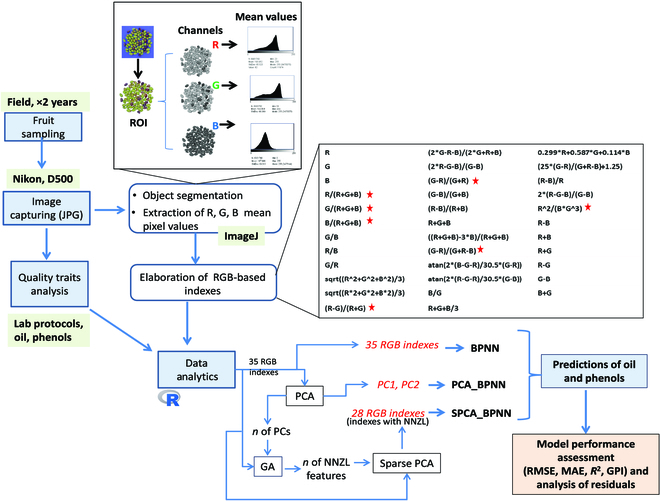
Flowchart of the experimental design for imaging, segmentation, fruit quality (oil and total phenols) determinations, and modeling through back propagation neural networks (BPNNs). After the segmentation, the region of interest (ROI) was sliced into R, G, and B channels (grayscale) and the mean of the light intensity distribution of the ROI in each channel was measured. The inputs (red text) of the BPNNs were the RGB-based colorimetric indexes (BPNN), the scores of the PC1 and PC2 resulting from the PCA (PCA_BPNN), and the RGB indexes having nonzero loadings (NNZL) selected after a Sparse PCA (SPCA_BPNN). The number of the PCs (2) and the original RGB indexes fed the genetic algorithm (GA) to determine the target of the number of features with nonzero loadings (NNZL) to be used as a input parameter of the Sparse PCA; the red star indicates the indexes not used for the SPCA_BPNN because after the Sparse PCA they had *loading* = 0.

Experiments were carried out in Southern Italy (Metaponto) during 2020 and 2021. The study area has a Mediterranean climate with mild winter and warm and dry summer; the details on the annual records of the main weather variables are reported in Fig. S1. The sampling time covered the period of oil and phenols accumulation lasting from pith hardening (end of July) till December. During this period, olive fruits were sampled at approximately 10-day intervals from three ~20-year old groves of the Coratina cultivar (5 × 6 m planting distance) and Frantoio and Leccino cultivars (6.5 × 6.5 m). Olive groves were irrigated and managed according to local commercial practices. In each field, 3 bulk samples (300 g each) were collected from 5 to 6 trees ensuring that olives were picked without petiole from the various exposure sides of the canopy. Samples were promptly transferred to the laboratory. Figure 1 reports the pipeline used in this work for imaging, segmentation, and data analytics.

### Image acquisition, processing, and data extraction

Each sample was partitioned into 3 subsamples of olives (about 100 g each) individually placed on the base of a stand holder covered with a blue paper as background (Fig. S2). A Nikon D5100 digital camera (16.9 Mpixels) was held to the stand to have the lens (AF-P DX Nikkor 18 to 55 mm, f/3.5-5.6 G VR, Nikon, Tokyo, Japan) positioned 45 cm away from the fruit. The stand holder was enclosed in a 0.8 × 0.8 × 0.8 m portable photo studio box (Ombar Photography Light Box) equipped with (light-emitting diode) LED 5500K, 100 LEDs on top of the box and sheltered through a light diffuser to avoid direct illumination of samples. Images were captured in JPG format and an X-Rite ColorChecker Classic color card (Grand Rapids, MI, USA) was used to ensure correct white balance and color. Namely, the color reference card was used to check repeatable conditions during image capturing across the various imaging sessions. Images were processed using the 1.53t version of ImageJ software [28]. Before RGB extraction, the “Image>Adjust>Brightness/contrast” command of ImageJ was used targeting the RGB coordinates of the white painted square of the color card to those of white (255, 255, 255). A total of 141 images (51 Coratina, 45 Frantoio, and 45 Leccino) were collected.

Images were segmented into fruit and background components (Fig. S2) by means of a macro calibrated by the user. Briefly, after the image was imported, it was processed to remove the background identifying the object of interest (olives); all the other objects were removed. Color thresholding for image segmentation starts through the Image>Adjust>Color Threshold command, choosing the default method within the L*a*b* color space, composed by lightness from black to white (L*), and the description of chromatic colors along the green–red (a*) and blue–yellow (b*) axis, respectively. The object of interest was selected, and selection coordinates were saved as the region of interest (ROI) within the ROI manager. After that, using the Image>Type>RGB stack command, the original imported image was converted into 3 layers (slices) stacked in a single window, with each slice representing the channel (grayscale) of the primary R, G, and B colors. The pre-saved ROI was pasted on each slice to select again the object of interest, and then the mean R, G, and B values were measured through the “measure RGB” plugin. A series of 35 colorimetric indexes were then calculated from the R, G, and B mean pixel values (Table 1).

**Table 1.** Colorimetric indexes used to predict the olive fruit quality traits. R, G, and B are the mean pixel values of the red, green, and blue color extracted from the image of the sample. In brackets, the source reference of the index.

Index	Formula	Index	Formula	Index	Formula
R		GLI	(2*G−R−B)/(2*G+R+B) [14]	GRAY	0.299*R+0.587*G+0.114*B [14]
G		HI	(2*R−G−B)/(G−B) [14]	GLAI	(25*(G−R)/(G+R−B)+1.25) [61]
B		NGRDI	(G−R)/(G+R) [18]	CI	(R−B)/R [62]
NR	R/(R+G+B) [15,61]	NDGBI	(G−B)/(G+B) [15,18]	SHP	2*(R−G−B)/(G−B) [62]
NG	G/(R+G+B) [15,61]	NDRBI	(R−B)/(R+B) [15,18]	RI	R^2/(B*G^3) [62]
NB	B/(R+G+B) [15,61]	I	R+G+B [18]	RminB	R−B [15,61]
GB	G/B [18]	S	((R+G+B)−3*B)/(R+G+B) [18]	RplusB	R+B [61]
RB	R/B [18]	VARI	(G−R)/(G+R−B) [14]	RplusG	R+G [61]
GR	G/R [18]	HUE	atan(2*(B−G−R)/30.5*(G−R)) [14]	RminG	R−G [15,61]
BI	sqrt((R^2+G^2+B^2)/3) [14]	HUE2	atan(2*(R−G−R)/30.5*(G−B)) [62]	GminB	G−B [15,61]
BIM	sqrt((R*2+G*2+B*2)/3) [62]	BGI	B/G [14]	BplusG	B+G [61]
SCI	(R−G)/(R+G) [14,15]	L	R+G+B/3 [62]		

### Fruit quality trait determination

After the imaging acquisition, each fruit sample was ground (skin + flesh + stone) into a paste with a hammer mill. About 75 g of well-mixed subsample of paste was used for a single determination of extractable fat matter (oil, % fresh weight), water content (%), and acidity (%) by near-infrared (NIR) analysis using the Olivia instrument (FOSS, Hillerød, Denmark). The subsample paste was spread on the sample cup and pressed to minimize the presence of air. The Olivia was operated with a single linear array detector (850 to 1,050 nm wavelength range) pre-calibrated by the factory and checked for zero before each measurement session after about 1 h warm-up.

Total phenols were determined on an aliquot of paste collected from the same subsamples used for NIR-base oil content determinations by the colorimetric reaction with Folin-Ciocalteu’s reagent. The phenols were extracted from a 5-g aliquot using 25 ml of 50% aqueous ethanol, and the resulting mixture was gently agitated for 24 h at room temperature. After centrifugation (15 min, 3,500 rpm) (Allegra 6R, Beckman Coulter 3750), an aliquot of 1.5 ml of the supernatant was drawn from the middle part of the tube using a 5-ml pipette. This sample was again centrifuged (10 min, 6,000 rpm) (minispin eppendorf f45-12-11) and a 500-μl aliquot was used for the determination of the total phenols. A 1-ml diluted (1:60, pure water) extract sample was added to 3 ml of Folin–Ciocalteu’s reagent (8.3%; v/v) and the mixture was vortexed for 10 s and allowed to stand for 6 min. Afterwards, 1 ml of 20% sodium carbonate (Na_2_CO_3_) solution was added with mixing. After 30 min at room temperature, the absorbance (abs) was read at 750 nm using a spectrophotometer (Varian 50-BIO, Varian Australia Pty Ltd, Victoria 3170 Australia). The total phenolic compounds were referenced to a standard curve (*y* = 0.0099*abs + 0.103, *R*^2^ =0.98) and reported as mg of gallic acid equivalents (GAE) per 1 g olive paste (skin + flesh + stone dry weight).

### Prediction of fruit quality traits

Oil and total phenols were modeled for each cultivar as response variables of RGB-based colorimetric indexes (covariates) using a resilient BPNN along with the logistic function as activation function[29,30]. The topology of the BPNN consisted of one input layer, one hidden layer, and one output layer. The output layer had one node, namely, the oil or total phenol concentrations. The BPNN had a single-hidden layer structure to reduce model complexity and minimize overfitting, the number of the nodes of the hidden layer was determined by the equation *N*/3 +2 according to Ref. [31], where *N* was the total number of input covariates.

Three BPNNs differing in the covariates of the input layer were used: (a) the RGB colorimetric indexes listed in Table 1 were used in “BPNN”; (b) the PCs determined through a PCA over those colorimetric indexes were used in “PCA_BPNN”; (c) a reduced number of the original colorimetric indexes were used in “SPCA_BPNN”. The input features were shifted to be zero centered and scaled to have unit variance before PCA. In addition, PCA was implemented with no limitation to the number of PCs to be used. The PC vectors to serve as input of the PCA_BPNN were determined using ~85% of total variance as cutoff point.

The input variables of the SPCA_BPNN were selected among the original RGB indexes as those variables having nonzero loadings after a sparse principal component analysis (SPCA) implemented through Ref. [32]. The RGB indexes used as input data of the SPCA (Table 1) were centered and scaled by subtracting the mean and dividing each by the standard deviation, hence having the data as per unit variance. The SPCA default maximum 200 iterations and 1*e*^−6^ quadratic penalty parameter were used. The number of variables with nonzero loadings to be achieved in each PC was specified through a GA according to Gajjar et al. [27]. The GA required the number of PCs to be considered. Hence, it used the resulting number of PCs determined through the abovementioned PCA.

Following a hold-out method, the entire number of observations in each cultivar (*n*) (51 Coratina, 45 Frantoio, and 45 Leccino) was randomly split into 2 parts: a training (70%) and a testing (30%) dataset. To account for the randomness of the training (and testing) dataset, this random subsampling was repeated 5 times (*j*), hence ensuring *j ≤ n* each time [33]. In each iteration, the 3 BPNNs topologies were fit to the same training dataset and evaluated on the same test dataset. The accuracy of each model was then assessed through the mean coefficient of determination (*R*^2^), the root mean squared error (RMSE), and the mean absolute error (MAE) as follows:

$$R^{2}=\frac{\sum_{i=1}^{n}{\left(X_{i}-\bar{X}\right)}^{2}{\left(Y_{i}-\bar{Y}\right)}^{2}}{\sum_{i=1}^{n}{\left(X_{i}-\bar{X}\right)}^{2}\sum_{i=1}^{n}{\left(Y_{i}-\bar{Y}\right)}^{2}},$$ (1)

$$\text{RMSE}=\sqrt{\frac{\sum_{i=1}^{n}{\left(Y_{i}-X_{i}\right)}^{2}}{n-1}},$$ (2)

$$\text{MAE}=\frac{\sum_{i=1}^{n}\left|X_{i}-Y_{i}\right|}{n}$$ (3)

where *X_i_* and $\bar{X}$ are the actual measured value and the mean of the measured values, respectively; *Y_i_* and $\bar{Y}$ are the estimated value and the mean of the estimated values, respectively; *n* represents the sample number of the estimated model.

The accuracy was also assessed in each BPNN model (*m*), through the general performance indicator (GPI) calculated over the 5 iterations (*j*) using the min–max (0,1) normalized values of the 3 (*i*) performance indicators *R*^2^, RMSE, and MAE according to [34]:

$${\mathrm{GPI}}_{m}=\sum_{j=1}^{5}\left[\alpha \;\left(\bar{O_{i}}\;-O_{\mathrm{ij}}\right)\right]$$ (4)

where α equals −1 for indicator *i* = *R*^2^ and 1 for all other indicators, and *O_i_* and $\bar{O}$*_i_* are the value and the median over the 5 iterations of the performance indicator, respectively. The main effect and interaction of year and stage on quality traits were evaluated by a 2-way analysis of variance (ANOVA). Prior to the ANOVA, the hypothesis of normality (Shapiro–Wilk’s test) and equal variance (Levene’s test) were tested (*P* < 0.05, SigmaPlot12.3; Systat Software, Inc. Palo Alto, CA, USA). These tests revealed the failure of the ANOVA assumptions. Hence, according to Kay et al. [35], data were aligned and rank transformed to allow a nonparametric ANOVA on a linear model *Y* ~ a*b +*c*, where the response variable was the oil or phenol concentration. The fixed effect terms were stage and year, respectively, and *c* was the random effect. Residuals of each single model were calculated as the difference between observed and fitted values. Residuals from the 5 iterations were pooled before the analysis conducted to evaluate their randomness and constancy of variance according to Ref. [36] and was implemented by means of Ref. [29].

## Results

### Seasonal variation of olive fruit quality traits

**Fig. 2.**
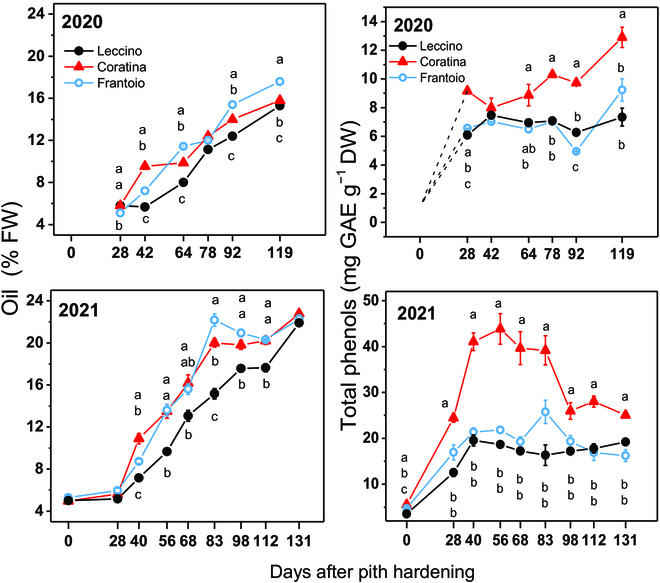
Seasonal trend of the mean (±SE) oil concentration (%FW) and total phenol concentrations (mg Gallic Acid Equivalent [GAE] per g [flesh + stone] DW) recorded in 3 olive cultivars during 2020 and 2021. Note that SE bars are visible when they are greater than the symbol. Within the same quality trait and year, and comparing between cultivars at the same time, different letters indicate statistically significant differences according to Tukey’s HSD test. Note that letters were not reported when there were no significant differences.

In both seasons, about 30 days after pit hardening (DAPH), the concentration of oil in fruit started to increase from a value of approximately 5.5%FW in all the cultivars reaching the maximum value of about 16%FW and 22%FW in 2020 and 2021, respectively (Fig. 2). The concentration values of the phenols differed between the 2 seasons in all cultivars, and the highest values were recorded in 2021. By contrast, the phenol concentrations in Coratina cultivar significantly differed from those of the Leccino and Frantoio for most of the sampling times in both seasons. Particularly, during the 2021 season, the initial increase in concentration peaked at approximately 41 mg GAE g^−1^ DW, thereafter it progressively decreased during the following months (Fig. 2). In addition to the significant effect of the cultivar, the analysis of variance revealed a statistically significant effect of the stage and of the year on oil and phenol concentrations (Table 2).

**Table 2.** Analysis of variance of aligned rank transformed oil (%FW) and phenol (mg GAE g^−1^DW) concentrations determined through a linear mixed model using the stage and year as factors. The 6 stages belong to the following 2020:2021 paired days after pit hardening: 28:28, 42:40, 64:68, 78:83, 92:98, and 119:112.

		*F*	Df	Df.res	Pr(>*F*)
Oil	Stage	170.7744	5	100	<2.22e−16 ***
	Year	156.8850	1	100	< 2.22e−16 ***
	Stage:Year	9.7429	5	100	1.3248e−07 ***

Phenols	Stage	2.6333	5	100	0.027964 *
	Year	309.9177	1	100	<2e−16 ***
	Stage:Year	2.97695	5	100	0.015107 *

Significance codes: ***, *α* = 0.001; * *α* = 0.05.

### Seasonal variation of R, G, B, and correlation between colorimetric indexes and quality traits

**Fig. 3.**
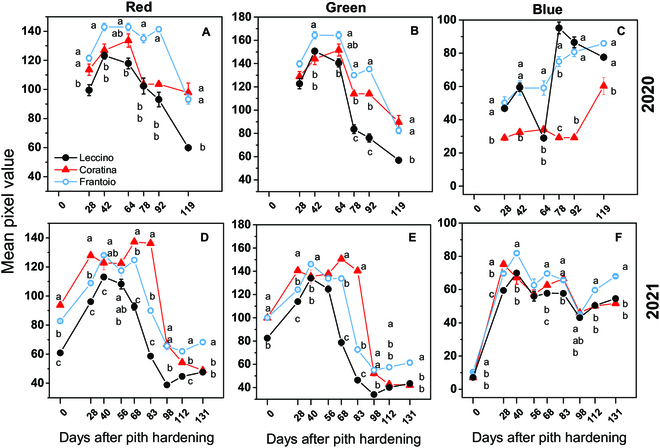
Seasonal trend of the mean (±SE) (A and D) red, (B and E) green, and (C and F) blue pixel values extracted from olive fruit images of the Coratina, Frantoio, and Leccino cultivars pictured during (top row) 2020 and (bottom row) 2021. Error bars are visible when they are higher than the symbol. Within the same color band and year, and comparing between cultivars at the same time, different letters indicate statistically significant differences according to Tukey’s HSD test (*P* value threshold 0.05). Note that letters were not reported when there were no significant differences.

The variations of the R, G, and B mean pixel values extracted from images during the 2020 and 2021 seasons are reported in Fig. 3. The patterns of the color bands were comparable across cultivars and years. Consistently in both years, the R (Fig. 3A to D) and G (Fig. 3B to E) mean pixel values initially increased toward the maximum values reached in all cultivars at approximately 40 DAPH. Such maximum values were somehow dependent on the cultivar. That is, the Leccino had the lowest R and G mean values in both years, while Frantoio had the highest R and G mean values in 2020. The R and G mean values remained at high levels for a variable time depending on the cultivar. Thereafter, the mean R and G pixel values declined toward the minimum, which was reached in advance in 2021 compared to 2020.

In contrast to R and G, the pattern of the mean B pixel values differed between the 2 years. That is, in 2020, the B pixels progressively increased in Leccino and Frantoio (excepting a transient decline at 64 DAPH in Leccino), while in Coratina, a steep increase was registered only at the end of the season (Fig. 3C). During 2021, after the initial steep increase, the B pixels decreased in all cultivars until 98 DAPH. Thereafter, B pixel values increased similarly to those in R and G (Fig. 3F).

**Fig. 4.**
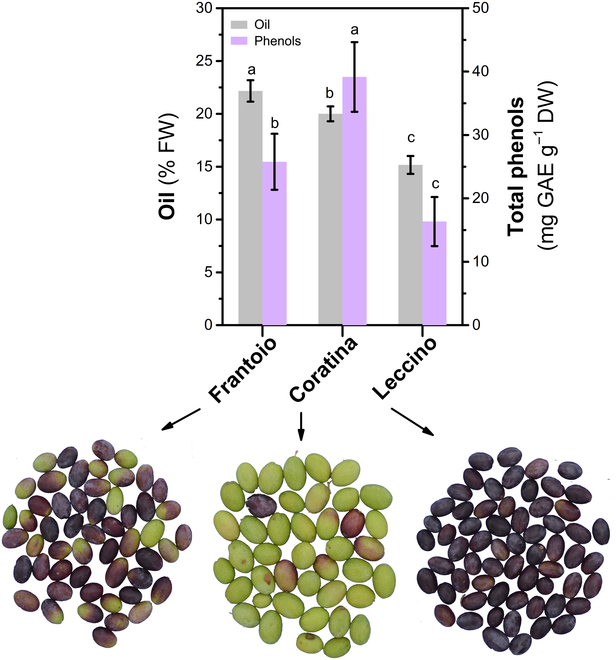
Mean (±SE) concentration of oil and total phenols in the fruit of Frantoio, Coratina, and Leccino olive cultivars measured at the same time point (at 83 DAPH, 2021 October 19). On the bottom, the corresponding olive samples after the image segmentation procedure. Comparing cultivars within the same variable, different letters indicate statistically significant differences according to Tukey’s HSD test at *P* value = 0.05.

**Fig. 5.**
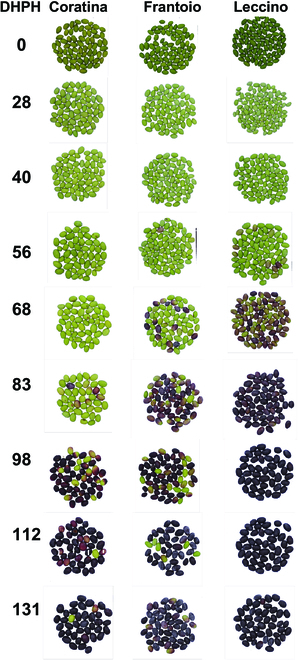
Example of some RGB images of the 3 cultivars captured during 2021. DHPH = days after pith hardening.

The pattern of the color bands (Fig. 3) and that of oil and phenols (Fig. 2) anticipate a differential relationship between phenotype and quality traits. For example, Fig. 4 shows the appearance of fruit along with their oil and total phenol concentrations determined at day 83 DAPH. The Leccino cultivar showed an advanced visual ripening (darker fruit) while having the lowest oil and phenol concentrations compared to the greener varieties (Frantoio, Coratina). The changes in the phenol concentrations were not linear as those of the oil ones (Fig. 2), making inferences based on visual appearance or linear correlation very difficult. An example of the seasonal variation of the phenotype of the 3 varieties is shown in Fig. 5.

**Fig. 6.**
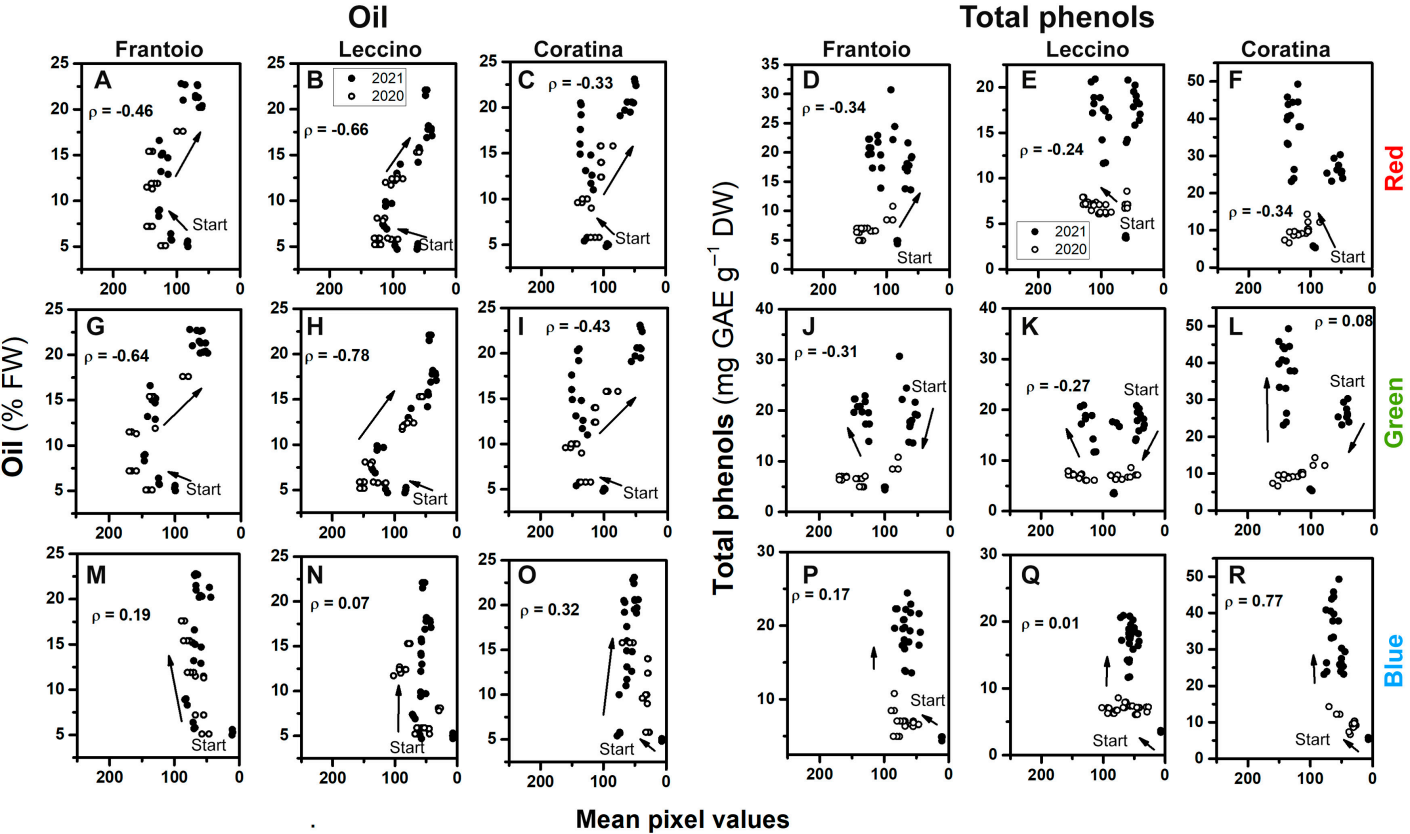
Correlation between the mean pixel values of image-based red (top row), green (middle row), blue (bottom row), and oil in (A, G, and M) Frantoio, (B, H, and N) Leccino, and (C, I, and O) Coratina cultivars, and total phenols in (D, J, and P) Frantoio, (E, K, and Q) Leccino, and (F, L, and R) Coratina cultivars, during (○) 2020 and (•) 2021. The arrows indicate the start and timeline of the samplings (from early to late), ρ = Spearman rank test coefficient of correlation.

Plotting the analyzed quality traits vs. the R, G, and B mean pixel values shows that data from the 2 seasons were consistent, and that the coefficients of correlation were variable (Fig. 6).

**Fig. 7.**
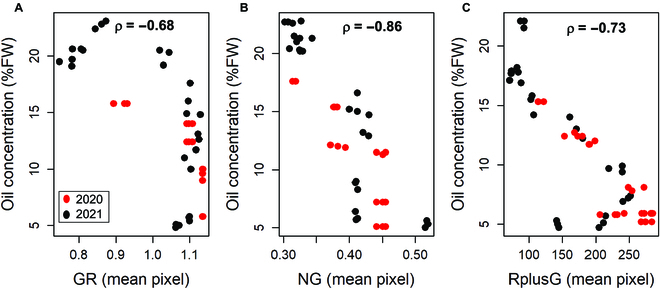
Example of correlation between oil concentrations and the mean pixel values of (A) GR = G × R in Coratina, (B) NG = normalized G in Frantoio, and (C) RG = R + G in Leccino. Data from 2020 and 2021 have been pooled before calculating the coefficient of correlation ρ (Spearman rank test).

**Fig. 8.**
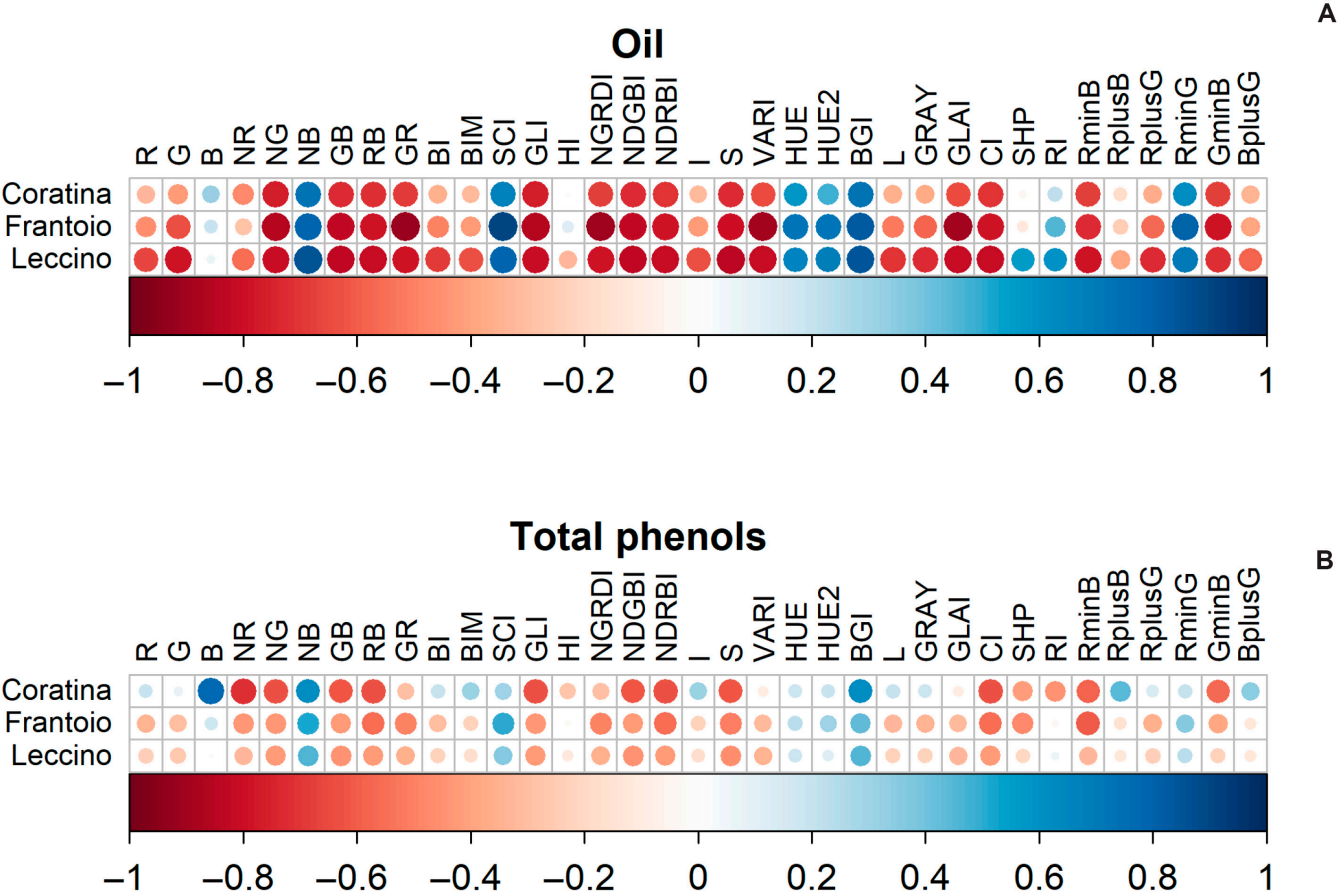
(A) Heatmap of the correlation coefficients (Spearman rank test) between the RGB-based colorimetric indexes and oil, and (B) total phenol concentrations determined in 3 cultivars.

Similarly, the changes in skin color as coded by the various new RGB-based colorimetric indexes were differentially correlated to the changes in the quality traits examined. For example, Fig. 7 reports the changes in oil concentration in parallel with the changes of G × R (GR), normalized G (NG), and R + G (RG) colorimetric indexes. All the resulting coefficients ρ (Spearman rank test) of the correlation between each quality trait and colorimetric indexes are reported in Fig. 8.

### Prediction of fruit quality traits

To predict the oil and the phenol concentrations, this study employed various ANNs differing in the input features. The BPNN used all the 35 RGB-based colorimetric indexes (Table 1). The resulting scores of PC1 and PC2 (Table 3) were used as input features of the PCA_BPNN. In the SPCA_BPNN, the input features were 28 of the abovementioned indexes, which were selected through the SPCA aided by a GA. The number of PCs used as input of the GA was 2, and it was determined through a standard PCA as the number of PCs whose cumulative percent of variance was close to 85% (see Fig. S3). Table 3 reports the output of the PCA and SPCA showing in bold the colorimetric indexes discarded (i.e., those with the loading value equal to zero) and not used as input of the SPCA_BPNN. Hence, the SPCA_BPNN used the indexes having the SPC1 with non-zero loadings. All the indexes of the SPC2 had zero loadings except the SHP, which was not considered because it was already deemed as an index with non-zero loading under the SPC1.

**Table 3.** Loadings of the 2 principal components determined by the PCA and by the sparse PCA (SPCA) over the RGB colorimetric indexes. The RGB indexes with nonzero loading of the SPCs were retained as input features of the SPCA_BPNN, while those in bold were excluded.

	PCA		SPCA			PCA		SPCA	
RGB index	PC1	PC2	SPC1	SPC2	RGB index	PC1	PC2	SPC1	SPC2
R	0.173673	−0.17527	0.190384	0	S	0.186705	0.150404	0.000897	0
G	0.193318	−0.13006	0.25154	0	**VARI**	0.192465	0.000261	0	0
B	−0.04634	−0.34902	0.029027	0	HUE	−0.19007	−0.0248	−0.00694	0
**NR**	0.112447	0.184999	0	0	HUE2	−0.16947	−0.01249	−0.00573	0
**NG**	0.195725	0.118161	0	0	BGI	−0.19762	−0.09568	−0.00164	0
**NB**	−0.18671	−0.1504	0	0	L	0.179046	−0.17924	0.457194	0
GB	0.107347	0.289417	0.001645	0	GRAY	0.184074	−0.16516	0.207812	0
RB	0.098209	0.290244	0.001023	0	GLAI	0.192465	0.000261	0.014478	0
GR	0.184904	0.037012	0.000741	0	CI	0.182407	0.13823	0.001129	0
BI	0.174901	−0.19118	0.172234	0	SHP	−0.01298	−0.01486	−0.00018	-1
BIM	0.162807	−0.22338	0.012636	0	**RI**	−0.1057	0.288167	0	0
**SCI**	−0.18633	−0.02744	0	0	RminB	0.194727	0.040558	0.160705	0
GLI	0.198202	0.105196	0.001438	0	RplusB	0.097349	−0.29776	0.208727	0
HI	0.009223	0.039836	−0.00091	0	RplusG	0.183643	−0.15131	0.436911	0
**NGRDI**	0.186327	0.027439	0	0	RminG	−0.18022	−0.00587	−0.05907	0
NDGBI	0.187861	0.155	0.001253	0	GminB	0.202835	0.031916	0.224126	0
NDRBI	0.170895	0.194543	0.000646	0	BplusG	0.141039	−0.25804	0.27192	0
I	0.161255	−0.22798	0.478908	0					

**Fig. 9.**
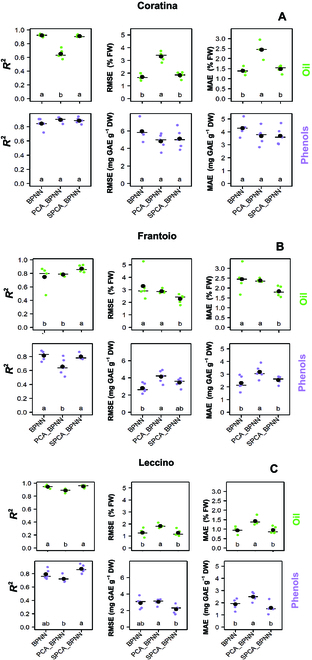
Point plot of the coefficient of determination (*R*^2^, left column) of the root mean squared error (RMSE, middle column) and of the mean absolute error (MAE, right column) retrieved after 5 iterations of the various BPNNs to predict oil (•, upper row) and total phenol (•, bottom row) concentrations in developing (A) Coratina, (B) Frantoio, and (C) Leccino cultivars. The same training and testing benchmark sets were used across the various BPNNs. Comparing the different BPNNs within the same parameter and cultivar, different letters indicate statistically significant differences at *P* < 0.05 (Kruskal–Wallis test). The horizontal line represents the median, and the biggest black dot represents the mean.

The various BPNNs were iterated 5 times on the same 5 randomly resampled subsets of the training and testing benchmark datasets. The outcomes of the models show that both the quality traits examined were accurately predicted with average values of the coefficient of determination (*R*^2^) ranging from 0.65 ± 0.03 (±SE) to 0.95 ± 0.01 and from 0.66 ± 0.05 to 0.9 ± 0.02 in oil and phenols, respectively (Fig. 9). Figure 9 also reports the accuracy parameters RMSE and MAE.

**Fig. 10.**
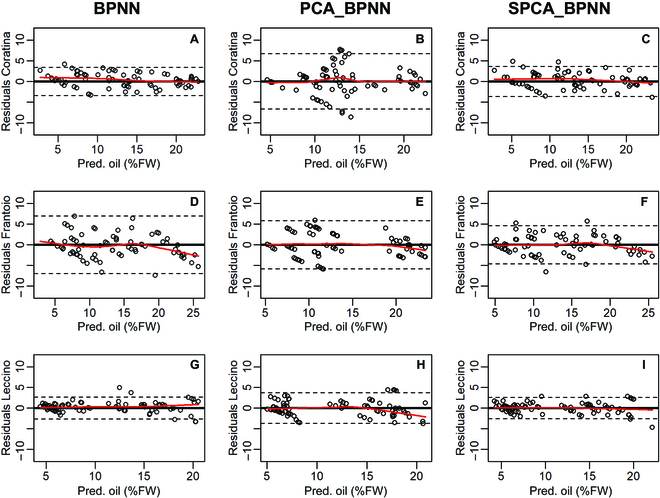
Residual plots of the BPNN, PCA_BPNN, and SPCA_BPNN models for the estimates of oil concentration in (A to C) Coratina, (D to F) Frantoio, and (G to I) Leccino cultivars. Residuals were calculated on the estimates resulting after 5 iterations of the various BPNNs 11 12; *n* = 75 in Coratina and Frantoio, *n* = 85 in Leccino. The bold horizontal lines represent zero in the ordinate values, dashed horizontal lines define the interval of the ±2 standard deviations calculated on the residuals, and the red line is the locally weighted scatterplot smoothing line.

**Fig. 11.**
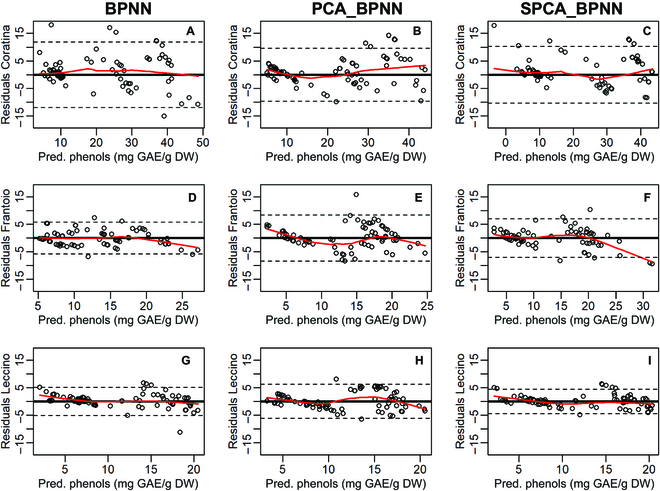
Residual plots of the BPNN, PCA_BPNN, and SPCA_BPNN models for the estimates of phenol concentrations (mg GAE g^−1^ DW) in (A to C) Coratina, (D to F) Frantoio, and (G to I) Leccino cultivars. Residuals were calculated on the estimates resulting after 5 iterations of the various BPNNs 12;*n* = 75 in Coratina and Frantoio, *n* = 85 in Leccino. The bold horizontal lines represent zero in the ordinate values, dashed horizontal lines define the interval of the ±2 standard deviations calculated on the residuals, and the red line is the locally weighted scatterplot smoothing line.

**Fig. 12.**
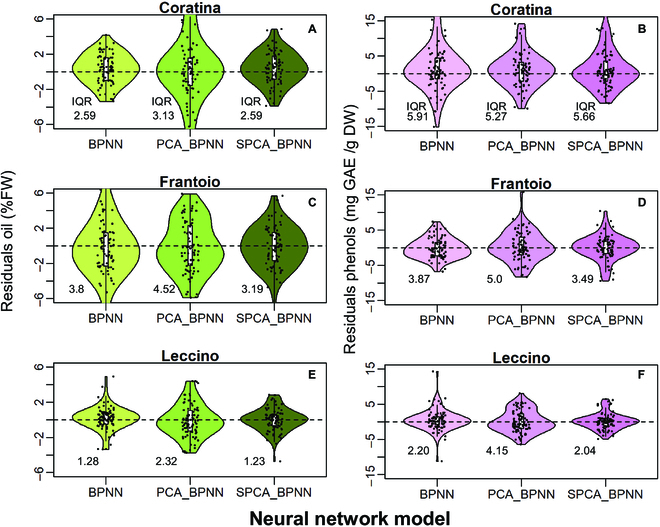
Distribution of residuals values (jittered dots) of the estimates of (A, C, and E) oil and (B, D, and F) phenol concentrations calculated through various BPNN models. The horizontal dashed line indicates 0. The interquartile range (IQR) is reported next to the distribution.

The constancy of variance of the residuals across the fitted oil and phenol concentrations is reported in Figs. 10 and 11. The variances of the residuals of oil data were homogenously distributed across the range of fitted measurements in most scenarios. However, the linearity of variance degrades mainly in Frantoio and Leccino for the higher oil concentrations (close to 25%FW) depending on the BPNN method (Fig. 10D, F, and H). Similarly, the variance of residuals of the phenols showed a non-linearity mainly for high values of concentrations (Fig. 11). Figure 12 presents the violin plots of the residuals for all BPNN models, cultivars, and quality trait scenarios. In every cultivar and consistently across oil and phenols, the SPCA_BPNN model had the skinnier shape of the residuals’ distribution and the narrower interquartile range (IQR) in almost all scenarios. By contrast, the distribution of residuals generated by PCA_BPNN had the larger IQR in 3/3 and 2/3 cultivars in oil and phenol concentrations, respectively. A bimodal shape was more evident for residuals belonging to the PCA_BPNN model. Detailed histograms and kernel density distributions are reported in Fig. S4.

The GPI values summarizing the overall prediction accuracies of the BPNNs are reported in Table 4. The GPIs were ranked differentially across cultivars and quality traits. Although it was not possible to systematically identify the best neural network model, it could be envisaged that the SPCA_BPNN had the highest number of first rank.

**Table 4.** Rank and values of the general performance indicator (GPI) calculated for the various back propagation neural networks (BPNN, PCA_BPNN, and SPCA_BPNN) used to predict the concentration of oil and total phenols in developing olive cultivars. The values in bold correspond to first rank.

GPI	Cultivar	BPNN		PCA_BPNN		SPCA_BPNN	
		Phenols	Oil	Phenols	Oil	Phenols	Oil
Rank	Coratina	2	3	**1**	2	3	**1**
	Frantoio	3	3	2	2	**1**	**1**
	Leccino	**1**	**1**	3	2	2	3
Values	Coratina	−0.15014	−0.1025	**0.17129**	0.21589	−0.17755	**0.37732**
	Frantoio	−0.41955	−0.51611	0.03186	−0.34027	**0.43254**	**0.17350**
	Leccino	**0.41724**	**−0.05665**	0.10131	−0.16239	0.17279	−0.62195

## Discussion

The present study documents affordable phenotyping prediction models of oil and total phenol concentrations in developing olive fruit using a BPNN fed with RGB-based colorimetric indexes extracted through imaging. The seasonal pattern of oil and phenol concentrations was tracked and modeled integrating current knowledge, which often focuses only on the endpoints of the fruit developmental period (e.g., harvest) and classification problems.

### Seasonal pattern of R, G, B, and fruit quality traits

The seasonal patterns of R, G, and B mean pixel values were quasi-parabolic if the curves of the B channel of the 2020 season are excepted. This result is difficult to discuss because of poor literature existing in this specific research field for olive and because fruit color is often empirically classified (e.g., [2,37]). However, the seasonal R, G, and B patterns agree with those recorded in grapevine [24]. Interestingly, although the curves of the R, G, and B channels of the various cultivars showed a similar shape, a significant shift in their values is displayed in most of the sampling times. Particularly, that shift was evident early in the season when the gain in quality traits of fruit had not started yet (Fig. 2) envisaging a putative genotype effect.

The oil concentrations increased throughout the season with a typical pattern reported for the Mediterranean area [5,38,39]. The different patterns of the RGB (non-linear) and that of oil concentrations (linear) anticipate that their correlation would be hysteretic. In addition, the coefficients of correlation span in a wide range, suggesting that the RGB indexes would have different weights as predictors. The phenol concentrations were variable between the 2 seasons and among the cultivars. The phenols, as well as oil, are influenced by cultivar [40]. Additionally, an inter-annual variability might occur due to environmental conditions and fruit load [39,41,42]. In line with this, Table 2 shows the statistically significant effect of *year* on the analyzed quality traits.

The fruit of cultivar Coratina showed the highest peak value of phenols compared to Frantoio and Leccino in line with the grouping criteria (high and low phenolic cultivars) suggested by Alagna et al. [40].

The early season increase of phenol concentrations corresponded to those of R, G, and B mean values; thereafter, R, G, and B declined, causing the hysteresis phenomenon and a weak linear correlation between them. It emerges that the single R, G, and B mean values were weak predictors of fruit quality traits within a linear correlation context. This would not be surprising considering that R, G, and B were not efficient even when used as input of a neural network to predict grapevine maturity [24]. However, after combining the R, G, and B values in new colorimetric indexes, most of them induced an improvement of the linear correlation coefficient according to Ref. [24]. In addition, a further improvement was observed when these colorimetric indexes served as input for the neural networks (see below).

### Structure of the neural networks

Image processing and neural networks have been employed in olive mainly to classify the fruit into specific ripening classes [43–46] or to sort them according to skin defects and color to support the production of olive oils with different quality [9,10]. In contrast, its use for regression is still limited. The present study would contribute to expanding the application of ANNs and imaging within a regression context in the olive sector covering the seasonal variation of the main fruit quality traits.

Application of ANNs might be constrained by the overfitting of models, an issue embedded in neural networks [26,46]. In the present study, a set of colorimetric indexes was derived from common R, G, and B values triggering the criticism that the derived indexes would be inter-related, and thus contributing to the overfitting of models.

However, the overfitting control in an ANN might be actively pursued through several methods including those based on PCA for the extraction of PCs or the selection of relevant features in sensu Bejani and Ghatee [26]. Accordingly, our study dealt with overfitting of models by adopting PCA pre-processing in PCA_BPNN and SPCA_BPNN.

The PCA is a multivariate technique used for the dimensionality reduction of the dataset with several quantitative dependent variables. The primary outcome of a PCA is a new set of ordered uncorrelated variables (PCs), which keep most of the variation of the original ones. There is a growing body of research using PCA pre-processing of datasets aimed at reducing the number of variables (e.g., ~350 or even 100,000) to a relatively small number of PCs (e.g., ~20) to be used as input of BPNNs [47]. The PCA performed over the original RGB-based colorimetric indexes showed that the first 2 PCs explained ~85% of the total variance, which falls in the common range (75% to 90%) of the cutoff point [48]. Hence, these 2 PCs were used as input for the PCA_BPNN. The performance of the BPNNs will be discussed in the next section.

In our study, the PCA pre-processing was employed to replace the colorimetric indexes with the scores of the PCs, and also to aid the selection of relevant colorimetric indexes (out of the total 35) to be used as input of the SPCA_BPNN. The loadings of PCA provide information about the relative importance of a variable to a PC. However, the interpretation of loadings aiding the selection of variables to be retained or discarded is not easy [27]. Hence, several techniques have been proposed to improve loading interpretability, including forcing them to be exactly zero, meaning the corresponding variables are not influential on PCs and therefore discarded (see Ref. [27] and references therein). Following this, the SPCA was employed in this study to identify the most important RGB-based variables, i.e., those with nonzero loadings. After the SPCA, the original 35 variables were reduced to 28, which were used as input of the SPCA_BPNN (see variables with nonzero loadings in Table 3).

In addition to the structure, an important feature of ANNs is their repeatability, which is influenced by the randomness of the test and training datasets [49]. Therefore, the 3 BPNNs have been iterated over the same 5 benchmark datasets, to ensure an unbiased comparison. However, the starting value of the weights for model initialization still represents a random component likely reducing the repeatability of models and their comparison. To face this issue, an algorithm-based initialization of models has been proposed in place of the random one, but with a minor improvement of the model accuracy [50]. Hence, the random component of weights initialization in our models would have been negligible, but this remains to be specifically tested.

### Prediction accuracy of the BPNNs

The modeling of the oil concentrations reached the highest mean *R*^2^ values of 0.92 ± 0.01 (±SE) (Coratina, BPNN), 0.87 ± 0.02 (Frantoio, SPCA_BPNN), and 0.95 ± 0.01 (Leccino, SPCA_BPNN) across the various ANNs (Fig. 9). Ram et al. [51] used 20 morphometric and 9 colorimetric fruit features as input of a neural network to predict the oil content in olive fruit. However, results showed a variable importance in the color-based features, which was ascribed by the authors to the late sampling period. In the present study, phenotyping of oil and phenols was implemented throughout almost the entire accumulation period, capturing a wide range of variation of the response variables (quality traits) and predictors (colorimetric features). A wide range is pivotal for model generalization and for the high predictive capability of a neural network in an actual scenario [52].

The prediction accuracy of phenols varied with the ANNs and showed the highest mean *R*^2^ values equal to 0.90 ± 0.02 (±SE) (Coratina, PCA_BPNN), 0.81 ± 0.02 (Frantoio, SPCA_BPNN), and 0.87 ± 0.03 (Leccino, SPCA_BPNN). This result is comparable to that reported for pulp samples of single olives predicted using portable NIR equipment [53]. The overall lower performance of BPNNs for prediction of phenols compared to that of oil could be explained considering the changes of colors of the inner pulp layers. These changes were not harvested by our contactless experiment and would be associated to changes of phenols. The image-based prediction of phenols collected only the changes in the color of the fruit skin. While using a destructive assessment method, the changes in color within the pulp layer are also recognized (i.e., Jaèn index) [37]. Hence, it would be interesting in a future study to account for this variation of color of the deep layers particularly for early ripening varieties (e.g., Leccino) having the veraison completed in advance compared to late ripening ones (e.g., Coratina) [2] while quality traits are still changing.

The effect of PCA pre-processing on the model predictions’ accuracy (*R*^2^) was variable. That is, the BPNN and SPCA_BPNN were comparably accurate when referring to the oil concentration in Coratina and phenols in Frantoio. At the same time, an improvement of the *R*^2^ was achieved when the SPCA pre-processing was employed in the case of phenol estimations in Coratina and Leccino cultivars and of oil in Frantoio and Leccino.

The PCA_BPNN showed the lowest *R*^2^ values across cultivars and quality traits if the phenols of the Coratina were excluded (Fig. 9).

However, the choice of statistics to evaluate the accuracy of neural network prediction models is still debated and there is a consensus that a single one would not collect the overall accuracy of the model. Particularly when various models have to be compared, because the coefficient of determination (*R*^2^) is highly sensitive to outliers, it is widely used in association with the scale-dependent RMSE and MAE [54,55]. In addition, the use of MAE would be encouraged as it is less sensitive to outliers than RMSE [56].

In our study, the PCA_BPNN tends to have a statistically significantly higher MAE and RMSE than the SPCA_BPNN, highlighting the reliability of the SPCA procedure in discarding non-relevant variables.

However, PCA-based BPNN improved the model’s prediction accuracy when the number of PCs retained as input was selected at a high cutoff (96% of total variance) [48]. Although SPCA pre-processing was intended to improve the PCA-based BPNN, it could be recognized that the SPCA_BPNN determined *R*^2^ values comparable to those of the BPNN or even better when the oil of the Frantoio cultivar was considered.

In order to provide an overall appraisal of the performance of the 3 ANN models, the values of *R*^2^, RMSE, and MAE were combined in a single GPI [34] similarly to an image-based study in grapevine [24]. In our study, the rank of the GPI reveals that the various BPNNs were inconsistently the best (or the worst) prediction models across the cultivars and quality traits. These results align with those in grapevine [24] and the recommendation in Ref. [51] to use cultivar-specific prediction models.

However, it could be noted that the SPCA_BPNN model ranked first in 2/3 (oil) and 1/3 (phenols) cultivars. In the case of the Leccino cultivar, it was always less accurate, ranking lower than the BPNN. The different ripening time of the cultivars can help to explain the variable rank of GPI. That is, globally, the Leccino and Coratina cultivars are considered early and late-ripening varieties, respectively [2,5]. The visual appearance of the fruit skin at the same time (e.g., Fig. 5) and the advanced decline of the R and G mean values in Leccino compared to Coratina (Fig. 3) confirm that these cultivars belong to a different ripening group. Hence, the inconstant GPI rank of predicting models suggests that a neural network with specific topologies might be designed for cultivars grouped according to their ripening period.

To improve model accuracy evaluation, graphical analysis of residuals has been proposed since the 1960s [36]. It was difficult to discuss our results due to the limited information existing on this specific research topic in olive. In our study, the stochastic characteristic of residuals and their homogeneity of variance across the range of the fitted values were examined. This revealed a different behavior between oil and phenols in terms of homogeneity of variance. For high values of oil concentrations, the linearity of variance degrades making the stochastic nature of the residuals criticizable. This suggests a differential ability of BPNN models to deal with the quality trait uncoupled from the phenotype, because oil continued to accumulate late in the season while RGB signals remained more stable (Figs. 2 and 3). The stage of the season had a statistically significant effect on the quality traits (Tab. 2), suggesting the introduction of a stage-based predictor into the model. This might improve the homogeneity of variance of residuals late in the season when oil concentrations are high. The non-linearity of the LOWESS (locally-weighted scatterplot smoothing) fitting curves confirms the heteroscedasticity of some distributions, which was anticipated by the violin and density plots (Fig. 12 and Fig. S4). However, the SPCA_BPNN and BPNN models had a roughly constant variance for Leccino (Fig. 11I), Coratina, and Leccino (Fig. 11A and G), respectively. Hence, more efforts are necessary to unravel the potential influence of a genotype-based factor if a unique model working for more than one cultivar is to be developed.

### Limitations

In this study, images were captured under laboratory standard conditions, whereas a direct imaging in an open field would have been influenced by variable conditions (e.g., light). Approaches that bring plant phenotyping from the lab to an open field to improve the reliability of a model are challenging [57]. Hence, more efforts are advisable before exploiting our findings. In this study, we employed RGB-based indexes because of their association with some intrinsic features related to olive quality traits (e.g., chlorophyll, phenols, and water content). This is in line with the idea that the successful use of image-based phenotyping to collect plant organ morphological and physiological traits would require the employment of an adequate proxy for image segmentation and processing [57]. However, the use of additional color spaces (e.g., L*a*b*) as suitable proxy of quality traits needs to be explored for a wider understanding in this specific research field.

The volume of data used in our study might potentially trigger criticism of the robustness of the method. In contrast with this, the volume is in the magnitude of those employed in other phenotyping studies, for example, to map the color of the berry in relation to genetic population variability and to quality traits ([24,58]). Importantly, the robustness of a predicting model is also related to its generalization capability. In line with this, the timing of the sampling procedure of our study has generated learning and validation datasets whose range almost entirely covered the variation of the colorimetric and quality traits. Thus, this would strengthen the generalization capability of each model [52,59].

The seasonal dynamics of fruit phenotype (i.e., skin color) in various growing areas might differentially be coupled with that of oil and phenols because of a variable gene × environment interaction. In this study, the genotype effect could be somehow envisaged by the significantly different R, G, and B channels observed in various cultivars at pith hardening (Fig. 3). However, the integration of the model with a parameter accounting for that interaction would be desirable. In addition, the putative genotype effect might contribute to explaining the fact that each variety has a different optimal ANN model. Furthermore, this suggests that the learning set of each cultivar conceivably belongs to a different domain of validity in sensu [59]. Although the estimation of the domain of validity of neural networks has inspired specific research for a long time (e.g., [59]), it remains challenging and was not targeted in our paper. However, it could be noted that “cultivar” resembles information of the “knowledge domain”, which should be provided to an ideal ANN working over several cultivars to improve its performance [60]. Hence, in such an ideal ANN, the cultivar would code for the change of the input feature (i.e., in our study, the RGB-based indexes, the scores of the PC1 and PC2, and the RGB indexes having nonzero loadings) to automatically switch to the best model.

Colorimetric indexes retrieved from RGB-based imaging of olive fruit were combined with a multilayer feed-forward neural network and applied as a phenotyping method to predict key fruit quality traits. The outcomes revealed that this method was satisfactorily able to predict oil and phenol concentrations in developing fruit. The use of 3 worldwide cropped varieties belonging to early, middle, and late ripening periods also revealed that a BPNN with specific topology might be designed for cultivars grouped according to their ripening period. The analysis of residuals claims future efforts to integrate the models with predictors accounting for genotype and stage effects in order to develop a unique model suitable at least for groups of cultivars. The use of RGB-based predicting models offered in this paper would favor an affordable phenotyping within the digital agriculture domain.

## Data Availability

The data are freely available upon request sent to the corresponding author.

## Supplementary Materials

Supplementary 1 Fig. S1. (A) Annual records of the daily mean temperature, evapotranspiration (*ET*_0_, Hargreaves method), and rainfall (bars) recorded during 2020 and (B) 2021 at the olive groves growing area (source: SAL Service, ALSIA Basilicata Region). Fig. S2. Example of some steps for the image-based data acquisition procedure with (A) raw image including the color reference card, (B) selection of the object of interest, and (C) segmented olive sample image, which will be processed for R, G, and B data extraction. Fig. S3. Results of the PCA conducted over all the 35 RGB-based colorimetric indexes. Fig. S4. Histograms of residual data values and kernel density curves (red line) determined for the estimates of (A) oil and (B) phenol concentrations in (left column) Coratina, (middle) Frantoio, and (right) Leccino cultivars using the various BPNN models; *n* = 75 in Coratina and Frantoio, *n* = 85 in Leccino.
