# Supplementary material for: Phenotyping Key Fruit Quality Traits in Olive Using RGB Images and Back Propagation Neural Networks
Source: Plant Phenomics. 2023 Jun 23;5:0061. doi: 10.34133/plantphenomics.0061 (PMC10289815; doi:10.34133/plantphenomics.0061)
Supplement: Supplementary 1 — Fig. S1. (A) Annual records of the daily mean temperature, evapotranspiration (ET0, Hargreaves method), and rainfall (bars) recorded during 2020 and (B) 2021 at the olive groves growing area (source: SAL Service, ALSIA Basilicata Region). Fig. S2. Example of some steps for the image-based data acquisition procedure with (A) raw image including the color reference card, (B) selection of the object of interest, and (C) segmented olive sample image, which will be processed for R, G, and B data extraction. Fig. S3. Results of the PCA conducted over all the 35 RGB-based colorimetric indexes. Fig. S4. Histograms of residual data values and kernel density curves (red line) determined for the estimates of (A) oil and (B) phenol concentrations in (left column) Coratina, (middle) Frantoio, and (right) Leccino cultivars using the various BPNN models; n = 75 in Coratina and Frantoio, n = 85 in Leccino. [file plantphenomics.0061.f1.docx]

Supplementary Materials

*Supplementary figures*


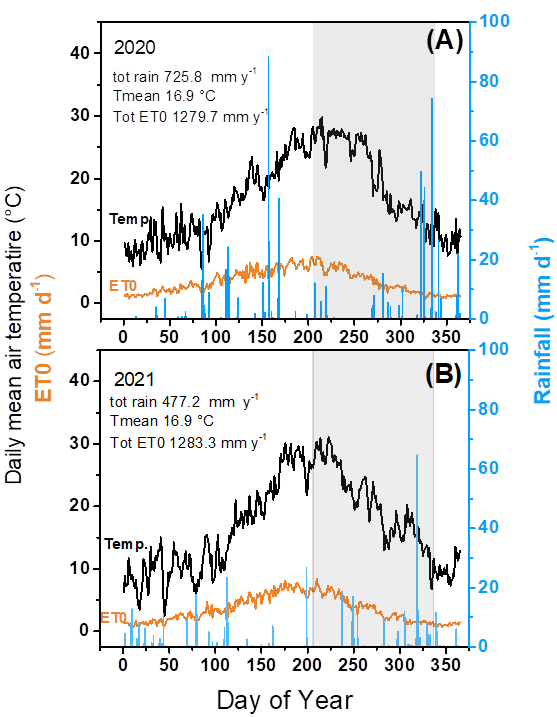


Figure S1: **(A)** Annual records of the daily mean temperature, evapotranspiration (*ET_0_*, Hargreaves method) and rainfall (bars) recorded during 2020 and **(B)** 2021 at the olive groves growing area (Source SAL Service, ALSIA Basilicata Region). The shaded area indicates the experimental period.


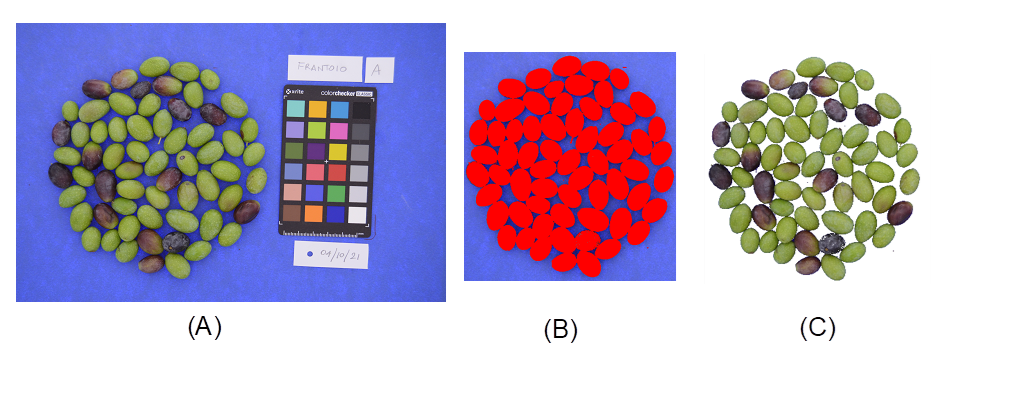


Figure S2: Example of some steps for the image-based data acquisition procedure with **(A)** raw image including the color reference card, **(B)** selection of the object of interest and **(C)** segmented olive sample image which will be processed for R, G, B data extraction.


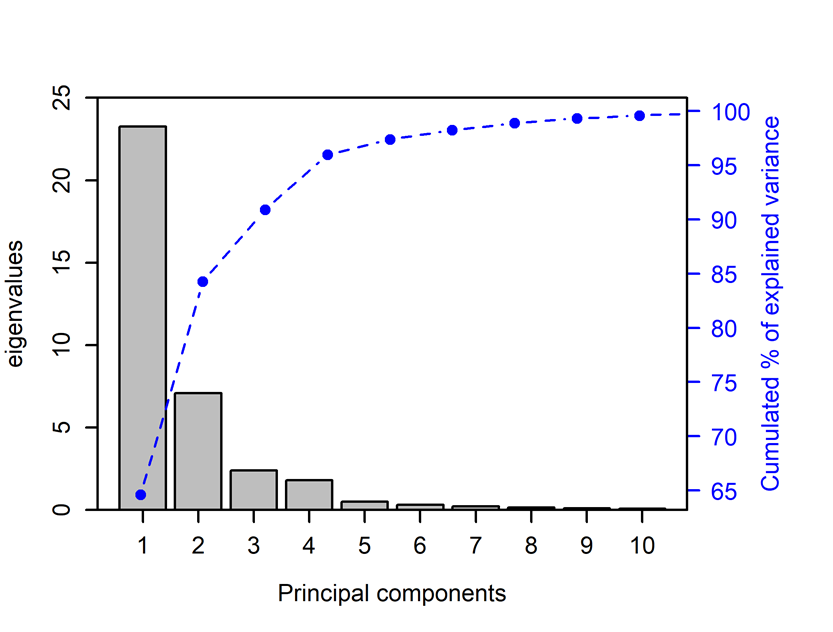


Figure S3: Results of the PCA analysis conducted over all the 35 RGB-based colorimetric indexes.

Figure S4: Histograms of residuals data values and kernel density curves (red line) determined for the estimates of **(A)** oil and **(B)** phenol concentrations in (left column) Coratina, (middle) Frantoio, and (right) Leccino cultivars using the various BPNN models; *n* = 75 in Coratina and Frantoio, *n* = 85 in Leccino.
